# BusyBee Web: towards comprehensive and differential composition-based metagenomic binning

**DOI:** 10.1093/nar/gkac298

**Published:** 2022-04-30

**Authors:** Georges P Schmartz, Pascal Hirsch, Jérémy Amand, Jan Dastbaz, Tobias Fehlmann, Fabian Kern, Rolf Müller, Andreas Keller

**Affiliations:** Chair for Clinical Bioinformatics, Saarland University, 66123 Saarbrücken, Germany; Chair for Clinical Bioinformatics, Saarland University, 66123 Saarbrücken, Germany; Clinical Bioinformatics (CLIB), Helmholtz Institute for Pharmaceutical Research Saarland (HIPS), Helmholtz Centre for Infection Research, 66123 Saarbrücken, Germany; Chair for Clinical Bioinformatics, Saarland University, 66123 Saarbrücken, Germany; Clinical Bioinformatics (CLIB), Helmholtz Institute for Pharmaceutical Research Saarland (HIPS), Helmholtz Centre for Infection Research, 66123 Saarbrücken, Germany; Microbial Natural Products (MINS), Helmholtz Institute for Pharmaceutical Research Saarland (HIPS), Helmholtz Centre for Infection Research, 66123 Saarbrücken, Germany; Deutsches Zentrum für Infektionsforschung (DZIF), Standort Hannover-Braunschweig, 38124 Braunschweig, Germany; Chair for Clinical Bioinformatics, Saarland University, 66123 Saarbrücken, Germany; Chair for Clinical Bioinformatics, Saarland University, 66123 Saarbrücken, Germany; Clinical Bioinformatics (CLIB), Helmholtz Institute for Pharmaceutical Research Saarland (HIPS), Helmholtz Centre for Infection Research, 66123 Saarbrücken, Germany; Microbial Natural Products (MINS), Helmholtz Institute for Pharmaceutical Research Saarland (HIPS), Helmholtz Centre for Infection Research, 66123 Saarbrücken, Germany; Deutsches Zentrum für Infektionsforschung (DZIF), Standort Hannover-Braunschweig, 38124 Braunschweig, Germany; Chair for Clinical Bioinformatics, Saarland University, 66123 Saarbrücken, Germany; Clinical Bioinformatics (CLIB), Helmholtz Institute for Pharmaceutical Research Saarland (HIPS), Helmholtz Centre for Infection Research, 66123 Saarbrücken, Germany

## Abstract

Despite recent methodology and reference database improvements for taxonomic profiling tools, metagenomic assembly and genomic binning remain important pillars of metagenomic analysis workflows. In case reference information is lacking, genomic binning is considered to be a state-of-the-art method in mixed culture metagenomic data analysis. In this light, our previously published tool BusyBee Web implements a composition-based binning method efficient enough to function as a rapid online utility. Handling assembled contigs and long nanopore generated reads alike, the webserver provides a wide range of supplementary annotations and visualizations. Half a decade after the initial publication, we revisited existing functionality, added comprehensive visualizations, and increased the number of data analysis customization options for further experimentation. The webserver now allows for visualization-supported differential analysis of samples, which is computationally expensive and typically only performed in coverage-based binning methods. Further, users may now optionally check their uploaded samples for plasmid sequences using PLSDB as a reference database. Lastly, a new application programming interface with a supporting python package was implemented, to allow power users fully automated access to the resource and integration into existing workflows. The webserver is freely available under: https://www.ccb.uni-saarland.de/busybee.

## Graphical Abstract

**Graphical Abstract:**
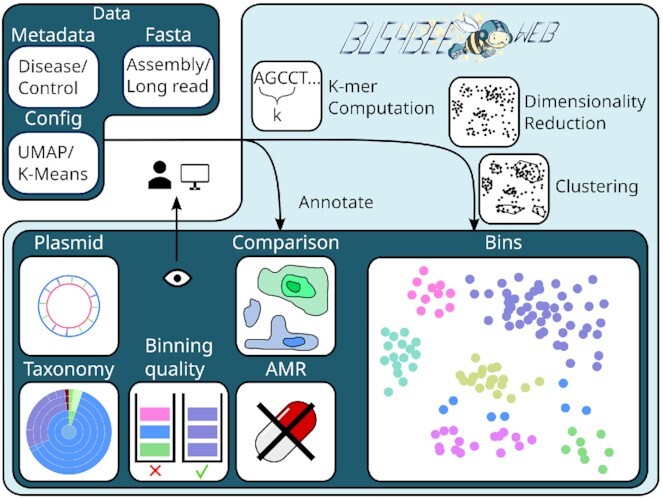
BusyBee Web is a flexible online composition-based and reference-free metagenomic binning tool. Sending fasta sequences as input via API or web interface, the webserver computes binned sequences and provides a wide range of optional annotations.

## INTRODUCTION

State-of-the-art metagenomics data analysis predominantly depends on reference databases. Reads are compared against well-characterized sequences and in case of sufficient sequence similarity, a read may be assigned to a taxonomy, an associated operational taxonomic unit count is incremented, or a genomic function is deduced (1–4). However, metagenomic studies operating at the boundary of what is known to humankind, e.g. investigating extreme maritime or volcanic environments, will inevitably come to the point where reference data is incomplete or of insufficient quality (5–7). While the overall possibilities for analysis are limited, a lack of reference information does not necessarily prevent any analysis. Instead, metagenomic short-read assembly or long-read metagenomic sequencing is frequently performed to allow for further hypothesizing, analysis, and discovery. However, due to high species diversity, sequencing errors, and other conflicts during assembly, metagenomic assemblies frequently yield multiple thousands of contigs of variable lengths and qualities (8,9).

Since short-read metagenomic read assembly and long-read metagenome sequencing output a mix of sequences of all the present species, structured analysis of results remains difficult. Therefore, longer sequences are usually grouped using binning methods to separate sequences into taxonomic units. Two features are frequently used to achieve informed separation into groups. Coverage-based binning uses coverage profiles of sequences, computed across multiple samples, to cluster into bins. Composition-based binning utilizes the conservation of sequence features like tetranucleotide profiles and derives bins from the input sequence (10). Many of the state-of-the-art binning methods such as MaxBin2 are hybrid methods using both kinds of features (11–13). However, coverage profiles provide limited information if only one individual sample is analyzed, and they may even be not applicable depending on the selected sequencing method. Accordingly, new methods that do not require coverage profiles are further developed (14,15).

In 2018, we proposed BusyBee Web as a reference-free composition-based binning tool efficient enough to function as a webserver (16,17). The underlying pipeline trains a classifier on a subset of the input data which is then used to assign sequences into bins. The used features are normalized k-mer profiles of length four or five. The tool optionally provides various functional and taxonomic annotations with Prokka and Kraken respectively allowing for taxonomic binning (18,19). Five years after initial publication, the community used BusyBee Web to analyze >2500 individual samples and perform >4500 runs. Here, we present a major update to the binning resource.

## MATERIALS AND METHODS

Developing an update to an existing resource allowed us to revisit some of the already available functionality and cover a broad list of minor improvements. Accordingly, the taxonomic annotation was updated to support Kraken 2 with a newer database and marker genes for bin quality assessment were extended to include the Archaea genes from the anvi’o project (20). Further, a sunburst plot was added and several new expert settings for clustering and embedding methods were implemented. Namely, we included t-SNE (21), Fit-SNE (22), UMAP (arXiv:1802.03426), PHATE (23) and TriMap (arXiv:1910.00204) as embedding and DBSCAN (24), HDBSCAN (25), *k*-means and spectral clustering (26) as new clustering methods. From the list of new features, we want to highlight three major changes with higher visibility to newer users.

### Plasmids annotations

Due to the random sampling involved in shotgun sequencing experiments, metagenomic data often includes plasmid fragments that may also end up in assemblies, potentially impacting downstream analysis. BusyBee Web now optionally compares input sequences to the most recent version of PLSDB using mash screen (27,28). In case plasmid signatures are found, the most relevant information about the plasmids is displayed. From here, users can take a deeper look into the findings by continuing their analysis on PLSDB.

### Comparative metagenomics

Group comparison is a frequently requested analysis that is often neglected in composition-based methods. In BusyBee Web, we compute a differential density between two user-defined classes, by first applying a Gaussian 2D kernel to the embedded sequences for both classes separately. Bandwidth and grid size used in the computation can be modified by the user, within given boundaries. Next, the difference between both densities is visualized. This usually results in a picture where various areas are dominated by different classes. While this method does not directly provide statistics on coverage differences, it remains indicative of different phenomena. On the one hand, if long reads are directly embedded, higher density regions should represent a higher relative number of sequences with a similar k-mer spectrum in the sample. On the other hand, if assembled contigs were provided, interpretation becomes more complex. First, the number of embedded sequences is expected to increase simply due to technological errors, resulting in higher density regions for higher sequence counts similar to the long-read interpretation. Second, increased phylogenetic diversity is captured since identical sequences should ideally be collapsed already during assembly. The difference in density can be retrieved for each cluster allowing the user to further analyze potentially interesting patterns and areas.

### Application programming interface

To allow programmatic access to BusyBee Web, we implemented an application programming interface (API). The API complies with the Open API 3.0.2 standard (29). Users can start jobs, check their status, and download individual results over the API. Additionally, a python package is supported and distributed via conda, which allows for easy integration into R scripts using reticulate. The package is available on: https://github.com/CCB-SB/busybee_api.

### Case studies

In order to benchmark BusyBee Web on a mock community in the first case study, we downloaded the *ERR3152364* dataset from the sequence read archive and converted the fastq files into fasta files while also adapting the header names. Due to the high sequencing depth of the experiment, the sample had to be pruned to comply with the constraints imposed by the webserver. Thus, we shuffled the fasta file randomly and selected the first 200 Mb of data, corresponding exactly to the upload limit and which accounts for <2% of the initial file. The resulting file contained a total of 50 679 reads. After data generation, we started analysis with default parameters changing only the embedding to UMAP. For comparison, various embeddings with different dimension reduction methods were computed (Supplementary Figure S1).

The second case study discussing differences between sequencing technologies was conducted with newly generated data. Both datasets were derived from the same 1mL of bile sample of a healthy human individual and DNA was extracted with the same QiAamp DNA Microbiome Kit allowing for comparison between technologies. Next-generation sequencing DNA libraries were prepared using the MGIEasy Universal DNA Library Prep set following the recommendations of the manufacturer. The DNBSEQ-G400 was used as short-read sequencing platform. Oxford nanopore sequencing was prepared with the SQK-LSK109 Ligation Sequencing kit before sequencing on an FLO-MIN106D flow cell in a MinION Mk1B. Basecalling was performed with Guppy v5.0.7. For both datasets, human-read contamination was removed by first running kneaddata v0.7.4, followed by sra-human-scrubber v1.0.2021_05_05 (1,30). After removal of human reads, the ONT fastq was converted to fasta and read names were shortened to generic header names. For the short-read sequencing data, reads were assembled to scaffolds with metaSPAdes v3.15.2 and scaffolds were retained (8). Before analysis with BusyBee Web, both datasets, short- and long-read, were combined and a mapping to the original fasta entries was generated. Next, data was passed to BusyBee Web with default settings, but selecting UMAP as embedding algorithm.

## RESULTS

With the increasing popularity of whole shotgun metagenome and long-read sequencing competing with amplicon sequencing, dedicated analysis of plasmids from metagenomics data is becoming increasingly tempting to the metagenomics community (31). However, shared sequences between chromosomes and plasmids, variable sizes, and a wide range of other factors render plasmid assembly from short reads an algorithmic challenging task often entailing high misassembly rates (31,32). Similarly, the prediction of both plasmid reads, and plasmid sequences remains an intensively debated field of research, also affecting long-read sequencing technology (33–38). Attributed to these difficulties, plasmid sequences frequently appear in binning inputs where they may be difficult to interpret. With the newly added plasmid annotation, BusyBee Web explicitly notifies the user about the presence of already known putative plasmid signatures. Further, the newly adopted differential density-based visualization allows for visual interpretation of similarity between aggregated samples. Since cohort and interventional studies comparing healthy against diseased patients, elderly against young, or different treatment conditions are increasingly performed in biomedical research, the field also faces an upsurge of comparative metagenomic studies. However, many of the conclusions drawn from cohort studies are either based on differential taxonomic counts or the functional aspect of sequences. In both cases, the comparison relies on reference information. One method to alleviate this constraint is to assess differential coverage profiles of binned sequences. However, similar to coverage-based binning, coverage profiles are required for this approach, which may not be available. Moreover, minor differences in binning outcomes may largely impact conclusions weakening the stability of this approach. The embedding followed by subsequent kernel application that we implemented alleviates these drawbacks and the volatility of results is bound to the characteristics of the selected dimensionality reduction method. Lastly, with the added application programming interface (API) BusyBee Web can easily be integrated into new and existing data analysis pipelines. In combination with workflow managing tools such as Nextflow or Snakemake, the API increases experiment throughput and reproducibility of results (39,40).

In order to highlight the improved functionality of BusyBee Web, we analyzed two datasets of varying ground truth information. While at the core BusyBee uses a reference-free algorithm for binning, here we make use of reference-based taxonomic annotations that were added after binning. Combining these annotations with the knowledge of well-characterized microbial environments allows us to better gauge binning quality.

### Mock community benchmark

**Figure 1.**
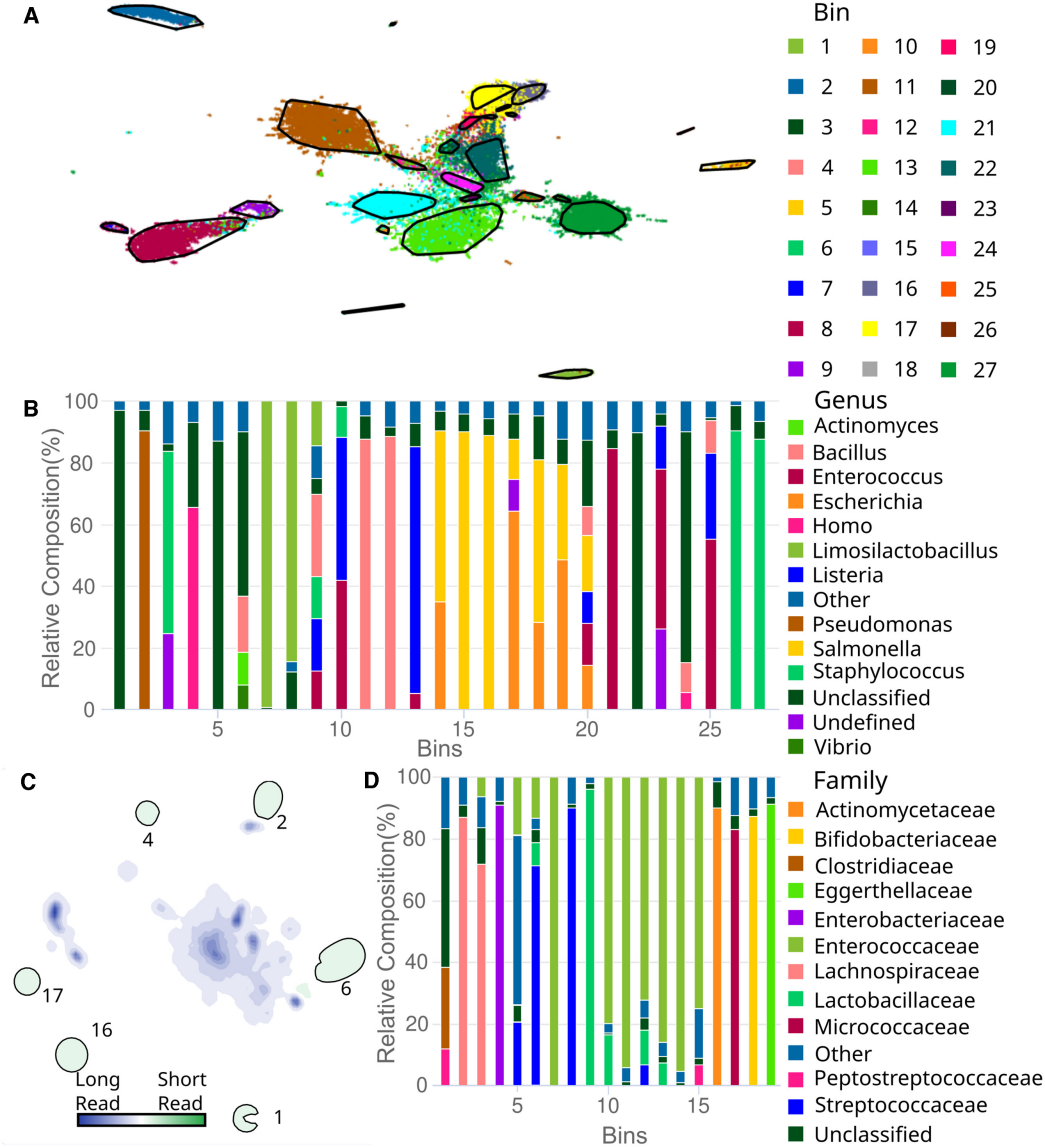
(**A**) Embedding of the mock community dataset, using UMAP with default settings. (**B**) Taxonomic profile at genus level of the different bins computed on a mock community composed of ten different species. (**C**) Differential density embedding of a bile sample sequenced with Oxford Nanopore MinION (Long Read) and DNBSEQ-400 (Short Read) respectively. (**D**) Taxonomic annotation of bins computed on the comparison dataset.

To assess the binning quality of BusyBee Web on a well-characterized example, we used a dataset by Nicholls et al. as ground truth (41). This nanopore sequencing data represents a mock community composed of exactly ten known species. The output of BusyBee Web consists of 27 bins (Figure 1A and B). However, 14 of these bins each contained <1% of sequences and may be discarded from further analysis. Of the remaining 13 bins, five bins, namely 1, 5, 8, 22 and 24, were mostly composed of unclassified sequences. We postulate that these bins are mostly made up of *Cryptococcus neoformans* and *Saccharomyces cerevisiae* which are not included in the selected Kraken 2 database. The taxonomic composition of bin 9, which is the smallest remaining bin composing only 515 sequences, is highly fractioned indicating a low binning quality. While not exempt from cross-contamination, all the remaining bins (2, 11, 13, 16, 17, 21 and 27) can clearly be attributed to the distinct species from the mock community, indicating that despite only using a fraction of the input, BusyBee Web is able to successfully recover the contained major species.

### Sequencing technology comparison

To highlight the new analysis functionality added in this update, we compared the suitability of long reads with short-read assembled scaffolds. With the 19,262 input sequences passing the default length filter a total of 19 bins were predicted of which five (5, 7, 8, 15 and 19) contained <1% of sequences. A total of 340 sequence similarities to potentially relevant plasmids were identified where the majority was reported in *Enterococcus faecium*. Looking at the new differential density plot from Figure 1C we observe six clusters (1, 2, 4, 6, 16 and 17) that are specific to the short-read sequencing experiment. The taxonomic profile of cluster 1 has a high relative number of unclassified sequences, pointing towards potentially unreliable assemblies (Figure 1D). Nevertheless, we note that within this bin a few long reads were found at a relative proportion of ∼15.5%. Four of the remaining five clusters (2, 4, 16 and 17) have low contaminations. These four clusters presumably consist mostly of Lachnospiraceae, *Enterobacteriaceae*, *Actinomycetaceae* and *Micrococcaceae* respectively. Potentially, due to biological random sampling or decreased sequencing depth, these genomic signatures mostly escaped the nanopore sequencing.

## CONCLUSION

With the new update, we substantially extended the capabilities of BusyBee Web as a versatile composition-based binning tool. On the one hand, with the newly added clustering methods, embedding algorithms, and API, we increased the data analysis possibilities for expert users. On the other hand, we hope to widen our user base by providing new visualizations and annotations. While we always strive for maximal flexibility, the ease of use of BusyBee Web as an installation-free webservice comes at a cost. For example, the data upload is limited to 200Mb per sample which can quickly be reached if multiple samples are being analyzed. Moreover, some of the presented clustering and embedding options will not be able to handle the theoretical maximal number of contigs that fit into a 200Mb file, due to time and memory constraints. Therefore, BusyBee Web provides an option for compressing information before embedding computation, alleviating some of these limitations. Nonetheless, visualization of the embedding in the local browser for many data points may become slow or irresponsive on less powerful hardware. Here, we recommend to prefer API usage instead. Moreover, with sufficient coverage information available, state-of-the-art coverage-based and hybrid metagenomic binning tools are expected to outperform composition-based tools on short-read sequencing data in larger projects.

Potential future development efforts may further focus on the identification of mobile genetic elements. However, with large disagreements already observed across plasmid classification tools, potential counter-strategies, e.g. automated removal of putative sequences from user input, are likely unstable and thus currently not advisable. Further, by extending the BusyBee Web server to allow for a selection of different embedding and clustering methods, it will be easier in the future to integrate newer algorithms into the generalized framework.

## DATA AVAILABILITY

BusyBee Web is freely available at: https://www.ccb.uni-saarland.de/busybee.

## ACCESSION NUMBERS

Respecting the German federal privacy law, we uploaded the short- and long-read data after human read removal to the Sequence Read Archive. Preprocessed data can be found in NCBI SRA using the accession numbers SRX14022915 and SRX14435297.

The mock community dataset was made available by Nicholls et al. in the Sequence Read Archive under the accession: ERR3152364.

## Supplementary Material

gkac298_Supplemental_File
