# Supplementary figures and images for: BusyBee Web: towards comprehensive and differential composition-based metagenomic binning
Source: Nucleic Acids Res. 2022 Apr 30;50(W1):W132–7. doi: 10.1093/nar/gkac298 (PMC9252796; doi:10.1093/nar/gkac298)

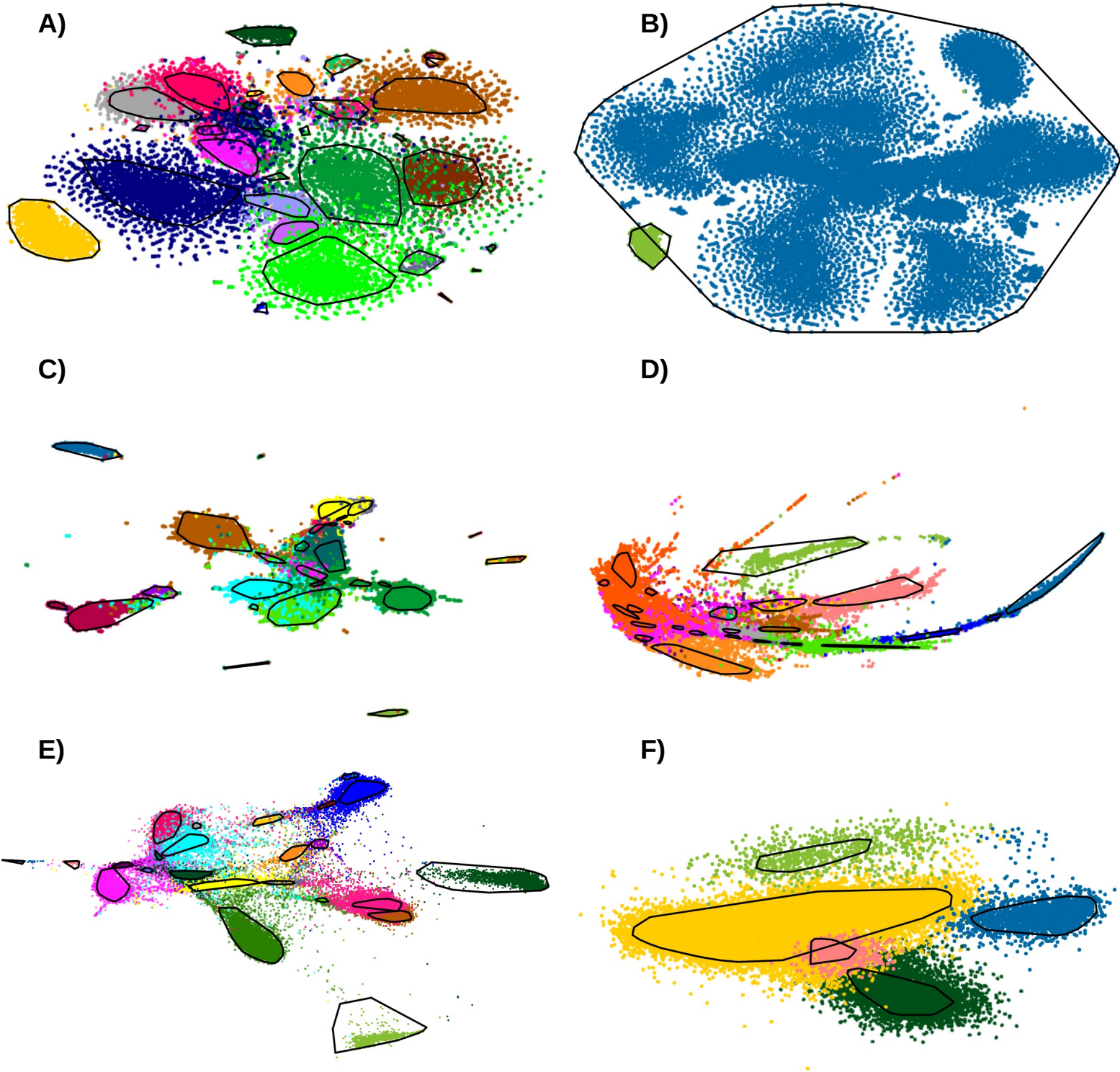

Supplement: gkac298_Supplemental_File [file gkac298_supplemental_file.pdf]
